# Cardiovascular Disease, Sleep-Disordered Breathing, and Artificial Intelligence: From Neutral Trials to Precision Sleep Cardiology

**DOI:** 10.31083/RCM50419

**Published:** 2026-07-28

**Authors:** Zhihua Huang, Jingjing Xiang, Zhihui Zhao, Qing Zhao, Anqi Duan, Zhaohong Sun, Lisheng Xu, Wenzhen Yao, Wenyu Zhang, Qi Wang, Luyang Gao, Xin Li, Yijia Wang, Sicong Li, Huiyi Liu, Chenhong An, Qin Luo, Zhihong Liu

**Affiliations:** ^1^Center for Respiratory and Pulmonary Vascular Diseases, Department of Cardiology, Fuwai Hospital, National Clinical Research Center for Cardiovascular Diseases, National Center for Cardiovascular Diseases, Chinese Academy of Medical Sciences and Peking Union Medical College, 100037 Beijing, China; ^2^Department of Information Center, Fuwai Hospital, Chinese Academy of Medical Sciences and Peking Union Medical College, 100037 Beijing, China; ^3^College of Information Science and Engineering, Northeastern University, 110819 Shenyang, Liaoning, China

**Keywords:** sleep-disordered breathing, cardiovascular disease, artificial intelligence, risk assessment, precision management

## Abstract

Sleep-disordered breathing (SDB), which includes both obstructive and central sleep apnea, is highly prevalent among patients with cardiovascular disease (CVD). Moreover, SDB contributes significantly to the development and progression of hypertension, coronary artery disease, arrhythmias, heart failure, and various cardiovascular and cerebrovascular events. However, despite strong mechanistic links involving intermittent hypoxemia, sympathetic activation, intrathoracic pressure fluctuations, and systemic inflammation, clinical trials of SDB treatment have yielded in neutral or even adverse cardiovascular outcomes. These results underscore the need for refined phenotyping, risk stratification, and personalized management. Artificial intelligence (AI) has emerged as a promising tool to address these challenges. In this review, we evaluate the mechanistic pathways through which SDB affects cardiovascular health and critically examine AI-based methods to enhance screening, outcome prediction, and treatment optimization. Applications include automated detection using clinical and biosignal data, cardiovascular risk prediction through machine-learning models based on sleep parameters, and AI-guided therapy personalization. Furthermore, we emphasize translational relevance by comparing model performance, identifying high-risk phenotypes, and exploring the potential for integration into clinical workflows. AI-enabled tools may help bridge the gap between pathophysiological understanding and improved outcomes by facilitating earlier diagnosis, tailored interventions, and proactive monitoring. Future studies should focus on prospective validation, regulatory pathways, and equitable deployment across populations.

## 1. Introduction

Sleep-disordered breathing (SDB), including primarily obstructive sleep apnea (OSA) and central sleep apnea (CSA), is a prevalent condition and is strongly associated with cardiovascular health. OSA alone affects approximately 1 billion individuals globally with approximately 24% of middle-aged men and 9% of women experiencing it [1,2]. A significant number of cases remain undiagnosed, and by some estimates fewer than 5% of moderate-to-severe cases of OSA are currently being diagnosed and treated [3]. Among those with cardiovascular disease (CVD), SDB is particularly common, and observational studies indicate that 40–60% of patients with CVD also have OSA [4,5,6], underscoring the clinical importance of OSA in this population.

OSA is characterized by repeated upper airway collapse during sleep, leading to apnea or hypopnea, oxygen desaturation, hypercapnia, and frequent awakenings [7]. These physiological events have both immediate hemodynamic and autonomic effects (such as spikes in blood pressure and heart rate during apnea episodes) and long-term consequences that increase cardiovascular risk. CSA, often presenting as Cheyne–Stokes respiration in heart failure (HF), predicts a worse outcome in these patients, and its presence is linked to significantly higher morbidity and mortality in cases of chronic HF [8]. This reciprocal relationship contributes to a considerable CVD burden and poses important clinical questions [9].

Nonetheless, although these issues with SDB are recognized, addressing them has not yet delivered the anticipated advantages in clinical trials on CVDs, highlighting the challenges associated with converting pathophysiological insights into improved clinical outcomes. For example, continuous positive airway pressure (CPAP), the primary treatment for OSA, significantly enhances daytime alertness and quality of life but did not notably decrease the incidence of major cardiovascular events in key studies [10,11,12]. The results of trials on CSA are even more concerning. An adaptive servo-ventilation (ASV) trial that enrolled patients with systolic HF and CSA was terminated prematurely because of an increase in cardiovascular mortality (10% annually compared to 7.5% in the control group) [13], leading to contraindications for ASV in patients with HF with reduced ejection fraction (HFrEF). These unexpected findings, wherein reducing sleep apnea did not clearly lead to fewer cardiac incidents—emphasize the significant gaps in our mechanistic understanding of these conditions.

**Fig. 1.**
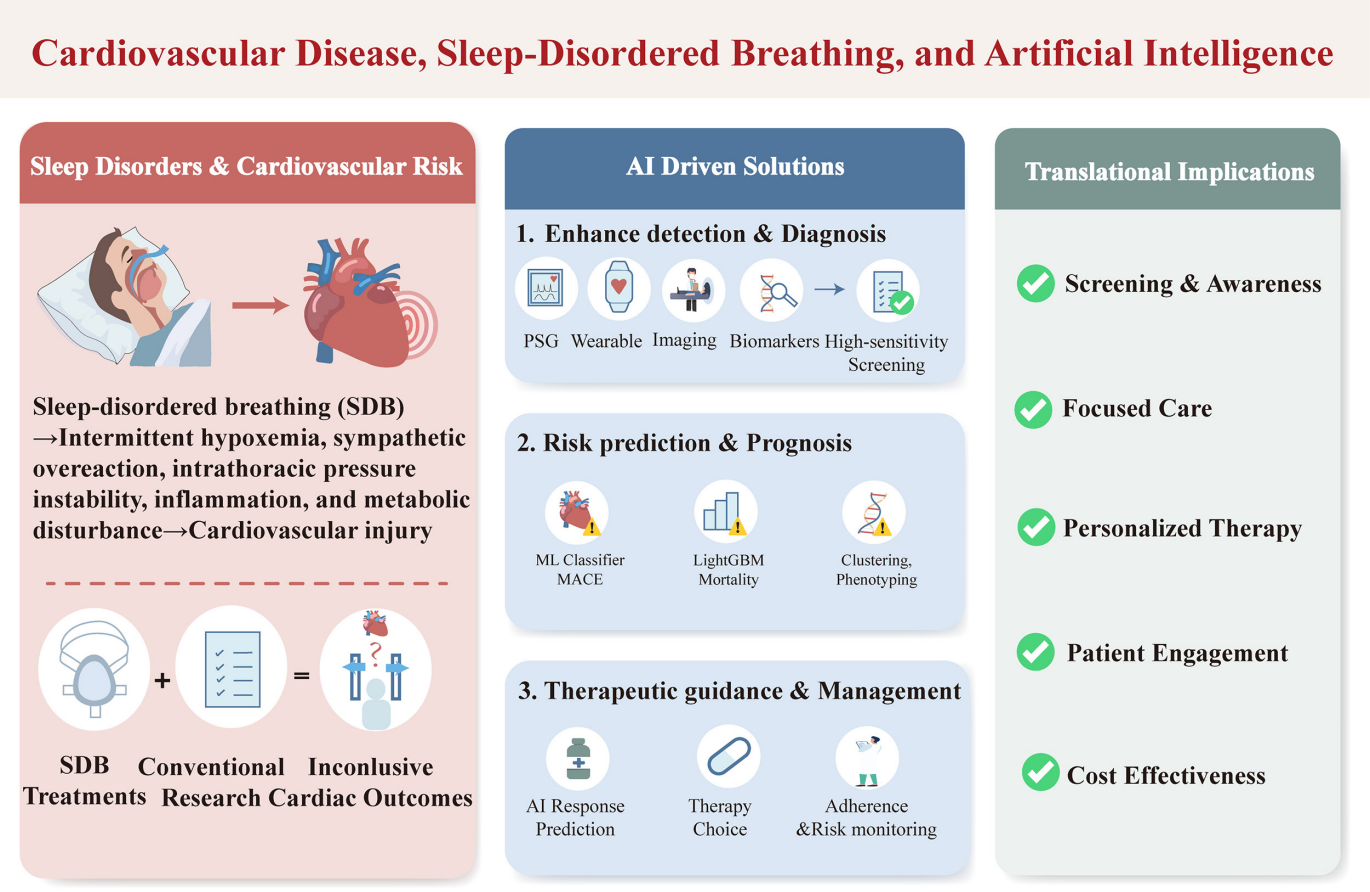
**Central illustration: overview of AI applications in SDB and cardiovascular disease**. While SDB contributes to cardiovascular injury through complex mechanisms, including intermittent hypoxemia and inflammation, conventional treatments often yield inconclusive results. This figure highlights how AI could help integrate detection, risk stratification, and personalized management to bridge the gap and improve clinical outcomes. PSG, polysomnography; ML, machine learning; MACE, major adverse cardiovascular events; AI, artificial intelligence; SDB, sleep-disordered breathing.

Artificial intelligence (AI) and machine learning (ML) have emerged as promising tools that may help to bridge some of the gaps at the intersection of sleep medicine and cardiology [14,15]. AI algorithms can help detect complex patterns in large datasets. In the context of SDB and CVD, AI applications range from enhanced screening and diagnosis to risk stratification, outcome prediction, and even therapeutic guidance. By integrating multi-modal data—such as physiological signals, imaging, laboratory test results, genomics, and clinical history, AI may uncover latent subphenotypes of OSA or CSA associated with different cardiovascular risks, enabling precision medicine approaches. In this article, we present a review of the recent findings at the intersection of CVDs, sleep disorders (with an emphasis on OSA and CSA), and AI (Fig. 1). We also highlight major clinical trials and studies to date, summarizing what they teach us about when treating SDB does (and does not) improve cardiovascular outcomes. Conceptual figures and tables are provided to distill key mechanisms, compare AI model performance, and provide an overview of landmark clinical trials. Finally, we discuss future directions, including research gaps, technical and regulatory challenges, and the path towards integrating AI-driven approaches into routine clinical cardiology and sleep medicine.

## 2. Literature Review

**Fig. 2.**
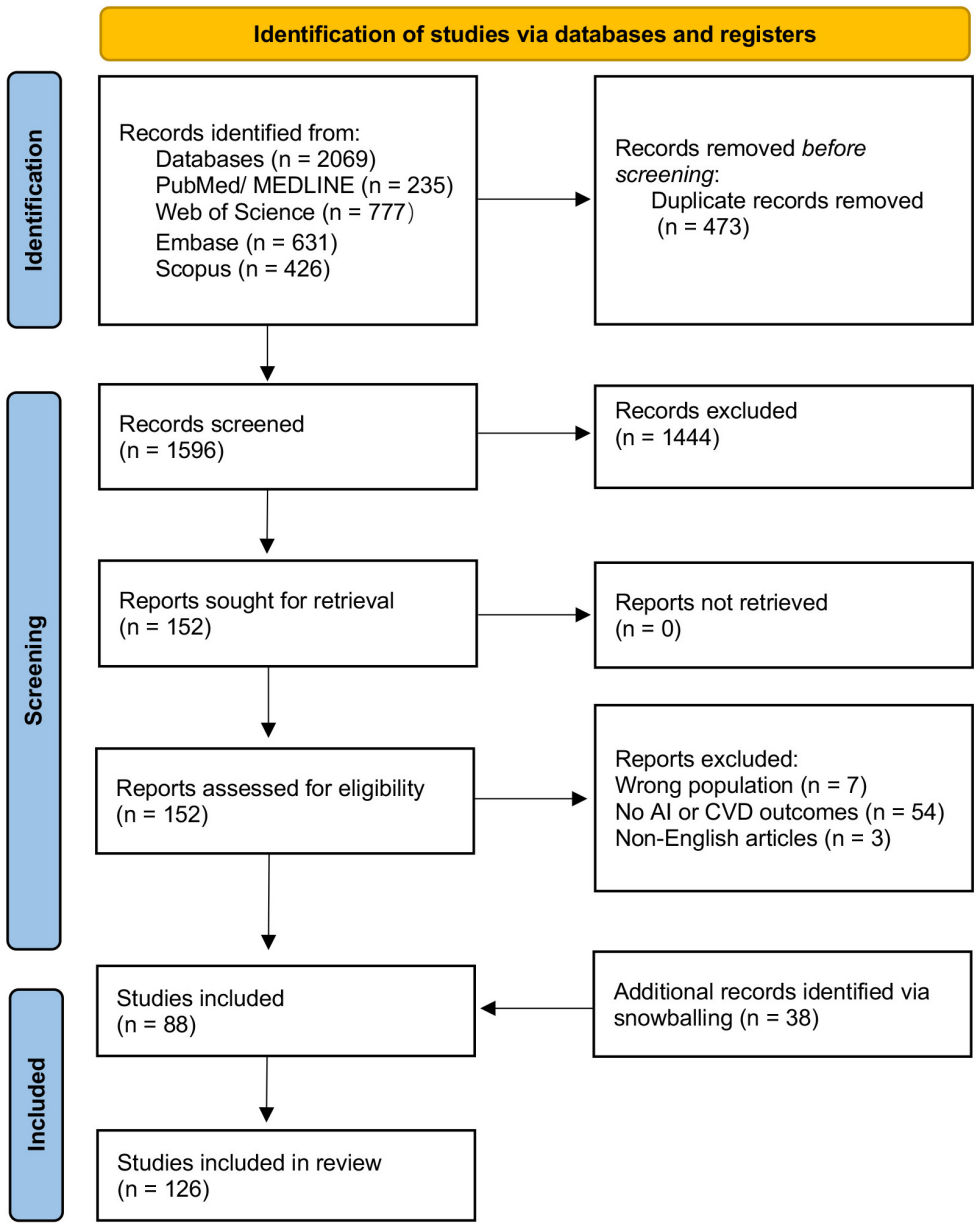
**PRISMA flow diagram of the study selection process**. This flowchart depicts the search and screening process of selecting studies of this review. A thorough search was performed across PubMed, Web of Science, Embase, and Scopus. Following the removal of duplicates and the exclusion of studies that did not meet the eligibility criteria, relevant articles were chosen for inclusion in the final analysis.

We conducted a systematic literature search to identify studies at the intersection of CVDs, SDB (notably OSA and CSA), and AI. The search strategy was developed in accordance with the Preferred Reporting Items for Systematic Reviews and Meta-Analyses 2020 guidelines to enhance the transparency and rigor of the study selection process. We examined multiple databases, including PubMed/MEDLINE, Embase, Web of Science, and Scopus databases, for English-language articles published up to March 2026. The search incorporated terms related to (1) sleep apnea (e.g., obstructive sleep apnea, central sleep apnea, sleep-disordered breathing, Cheyne-Stokes respiration), (2) cardiovascular conditions (e.g., hypertension, coronary artery disease, heart failure, atrial fibrillation, and stroke), and (3) AI techniques (e.g., machine learning, deep learning, neural network, artificial intelligence, and predictive models). Our search initially identified 1596 records after deduplication. Following the title and abstract screening, 152 studies were selected for full-text review. An additional 38 studies were identified through manual snowballing from key reviews and reference lists. Of these, 126 studies met all the criteria and were included in the qualitative synthesis (Fig. 2).

## 3. Pathophysiological Links Between SDB and CVD

**Fig. 3.**
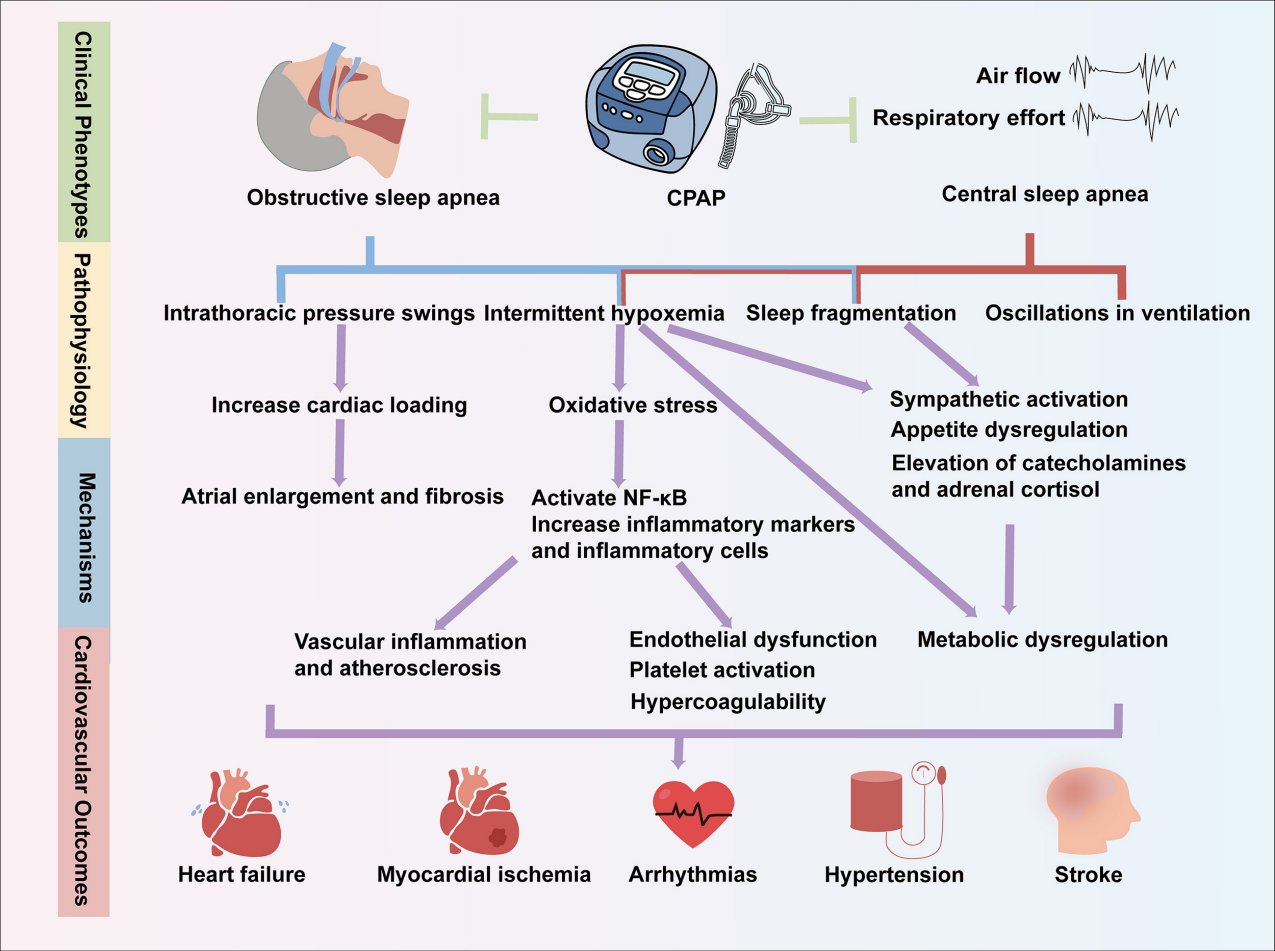
**Pathophysiological mechanisms linking sleep apnea to cardiovascular outcomes**. Both obstructive and central sleep apnea trigger a cascade of physiological changes, including intermittent hypoxemia, fluctuations in intrathoracic pressure, and sleep fragmentation. These alterations result in oxidative stress, sympathetic activation, inflammation, endothelial dysfunction, and metabolic dysregulation. These mechanisms contribute to cardiovascular outcomes like heart failure, myocardial ischemia, arrhythmias, hypertension, and stroke. CPAP may help mitigate these effects in sleep apnea by addressing upstream pathophysiological disturbances. CPAP, continuous positive airway pressure; NF-κB, nuclear factor-κB.

Both OSA and CSA have complex effects on the cardiovascular system, involving immediate physiological stresses as well as prolonged neurohumoral changes. Fig. 3 depicts the primary mechanisms by which sleep apnea can contribute to CVD.

### 3.1 Intermittent Hypoxemia and Oxidative Stress

The characteristic oxyhemoglobin desaturations in OSA, and to a lesser extent in CSA, initiate cycles of hypoxia-reoxygenation injury. This process leads to the production of reactive oxygen species and oxidative stress in vascular tissues [16], which subsequently activates pro-inflammatory transcription factors such as nuclear factor-κB and downstream inflammatory cascades, thereby promoting vascular inflammation and atherosclerosis [16]. Repeated hypoxia also upregulates hypoxia-inducible factors, contributing to dyslipidemia and insulin resistance, and further increasing cardiometabolic risk.

### 3.2 Sympathetic Activation and Hemodynamic Stress

Each apnea episode triggers a surge in sympathetic nervous system activity owing to hypoxemia and arousal-related reflexes, leading to transient nocturnal blood pressure spikes and vasoconstriction. Over time, these episodes result in sustained daytime sympathetic overactivity and hypertension [17]. The chronic elevation of catecholamine levels in OSA contributes to left ventricular (LV) hypertrophy, arrhythmogenicity, and vascular remodeling, thus serving as a key mechanism linking OSA to CVD [6]*.* Elevated resting heart rate and reduced baroreflex sensitivity in patients with OSA reflect this autonomic imbalance. Moreover, OSA independently increases the risk of developing hypertension, and treating OSA can modestly lower blood pressure [18,19]. Sympathetic surges also predispose individuals to cardiac arrhythmias; for instance, apnea-induced increases in heart rate and blood pressure can trigger atrial ectopy and atrial fibrillation (AF) in susceptible individuals [20,21].

### 3.3 Intrathoracic Pressure Swings and Cardiac Loading

OSA is marked by vigorous inspiratory efforts against a blocked airway, often resulting in significantly negative intrathoracic pressure that can be as low as −50 mmHg. These fluctuations in intrathoracic pressure substantially increase the transmural pressure stress on the heart [22]. During apnea, sudden negative pressure boosts venous return and right ventricular (RV) preload while simultaneously raising the LV afterload. This leads to acute myocardial stretch and septal shift, which can temporarily decrease cardiac output and trigger arrhythmias. Over time, these repetitive pressure fluctuations contribute to atrial enlargement and fibrosis, creating an arrhythmogenic substrate that likely explains the strong association between OSA and AF [5,21]. Additionally, negative pressure intermittently increases LV wall stress, potentially accelerating HF progression in individuals with compromised systolic function [23]. In summary, the distinct mechanical stresses of OSA, which are absent during normal breathing, impose a hemodynamic burden on the heart and vasculature.

### 3.4 Sleep Fragmentation and Neurohormonal Changes

Recurrent arousal during sleep results in fragmented sleep architecture and a loss of restorative deep sleep. This fragmentation activates the sympathetic-adrenal axis and impairs vagal tone, contributing to daytime hypertension and insulin resistance [24]; it also increases adrenal cortisol secretion, linking OSA to features of metabolic syndrome. Poor sleep triggers appetite dysregulation through changes in leptin and ghrelin levels, leading to weight gain and potentially exacerbating obesity and a self-perpetuating cycle, as obesity is a major risk factor for OSA [16]. Furthermore, patients with OSA often exhibit abnormal blood pressure patterns, such as non-dipping or even rising blood pressure overnight, which is associated with a higher cardiovascular risk [25]. The levels of endothelin-1, a potent vasoconstrictor, are elevated in OSA and improve with CPAP therapy, indicating endothelial activation due to apnea stress [26].

### 3.5 Inflammation and Endothelial Dysfunction

Patients with OSA exhibit increased levels of circulating inflammatory cells and endothelial dysfunction, as indicated by impaired flow-mediated dilation measurements [16,27]. Intermittent hypoxemia leads to the upregulation of adhesion molecules including intercellular adhesion molecule-1 (ICAM-1) and vascular cell adhesion molecule-1 (VCAM-1), which attract leukocytes to vessel walls and promote the oxidative modification of low-density lipoprotein (LDL), thereby encouraging plaque formation [28]. OSA is also linked to enhanced platelet activation and hypercoagulability (e.g., increased fibrinogen, factor VII, and plasminogen activator inhibitor-1 [PAI-1] levels) [6,16], particularly in the morning following apnea. This may account for the increased risk of nocturnal myocardial infarction and stroke in these patients, with the combination of hypercoagulability and endothelial injury creating an environment conducive to thrombotic cardiovascular events.

### 3.6 Metabolic Dysregulation

OSA can induce or exacerbate insulin resistance, glucose intolerance, and dyslipidemia, partly because of the effects of intermittent hypoxia on adipose tissue and liver metabolism. The sympathetic surges and sleep deprivation associated with OSA promote hyperglycemia and reduce insulin sensitivity [29]. Over time, untreated OSA has been associated with the onset of type 2 diabetes, and this metabolic burden further increases the risk of coronary artery disease and cardiomyopathy. It is important to note that CSA in HF involves somewhat different dynamics but results in similar stressors. In CSA, oscillations in ventilation (hyperventilation-apnea cycles) lead to fluctuations in blood gas levels and arousals, but without large intrathoracic pressure swings (because breathing effort is reduced in central apneas). Nevertheless, CSA still causes intermittent hypoxia, sympathetic surges, and sleep fragmentation. In systolic HF, Cheyne-Stokes respiration is driven by an unstable respiratory control feedback loop (high chemosensitivity and prolonged circulation time). The presence of CSA reflects severe circulatory compromise and induces further sympathetic activation and arrhythmias [30]. In fact, patients with HFrEF and CSA have high mortality, and CSA with a Cheyne-Stokes pattern is a strong marker of advanced HF and is associated with a significantly increased risk of death [31,32]. Thus, although OSA and CSA arise from different primary mechanisms (airway collapse vs. unstable ventilatory control), both can worsen cardiovascular function through hypoxemia, adrenergic stimulation, blood pressure variability, and inflammatory stress.

Understanding these mechanisms is critical, as they form the rationale for the expectation that treating sleep apnea could prevent cardiovascular events. Disappointing trial results, such as those of the Sleep Apnea CardioVascular Endpoints (SAVE) trial, indicate that timing and patient selection are critical, as OSA may be detected after irreversible injury has occurred in many patients with established CVD. Alternatively, not all cases of OSA are comparable—some patients have more predominant hypoxic stress whereas others have more arousal-heavy OSA, and the former may carry greater CV risk (“malignant OSA”) while the impact in the latter case is more metabolic or neurocognitive. This raises the notion of OSA subphenotypes or “endotypes” that may differentially drive cardiovascular injury. As discussed in later sections*, *AI methods are especially well-suited for exploring such distinctions in large datasets.

## 4. Clinical Impact of Sleep Apnea on Cardiovascular Outcomes

Before exploring AI solutions, we first summarize the clinical evidence linking SDB to cardiovascular outcomes, drawing from both observational data and interventional trials, to identify areas for potential improvement. Epidemiological and cohort studies have consistently shown that patients with sleep apnea experience higher rates of cardiovascular events over time than those without the condition, even after adjusting for confounding factors. For instance, a landmark longitudinal study by Marin et al. [33] tracked men for over a decade and found that untreated severe OSA was associated with a significantly higher incidence of fatal myocardial infarction and stroke than those in healthy sleepers or individuals treated with CPAP. The Sleep Heart Health Study [34], a large community-based cohort study, demonstrated that OSA severity correlates with the risk of coronary heart disease and stroke in middle-aged and older adults, independent of common risk factors. OSA is also highly prevalent among patients with resistant hypertension [35] and those with AF [36], suggesting that it plays a role in the development or maintenance of these conditions. Notably, among patients with AF undergoing catheter ablation, those with untreated OSA have significantly higher rates of AF recurrence, whereas OSA treatment is associated with improved arrhythmia-free survival, providing evidence that OSA treatment can modify arrhythmic outcomes in this context [37].

However, the results of randomized trials aimed at treating OSA to enhance cardiovascular outcomes have been predominantly neutral. For example, the SAVE trial (2016), which included patients with moderate to severe OSA and established CVD (primarily prior stroke or myocardial infarction), found no significant reduction in composite cardiovascular outcomes after CPAP therapy [12]. Most participants were asymptomatic for OSA, and the average CPAP usage was 3.3 h per night. Notably, CPAP improved subjective well-being and mood, reinforcing its known benefits for quality of life. However, for “hard” outcomes such as recurrent stroke or heart attacks, CPAP showed no difference over approximately four years on an intention-to-treat basis. Post hoc analyses suggested that patients using CPAP for ≥4 h per night may have experienced better outcomes, but adherence was suboptimal in many cases. Similarly, the Randomized Intervention with CPAP in CAD and OSA (RICCADSA) trial [11], which includes patients with coronary artery disease and minimally symptomatic OSA, found no significant differences in outcomes between the CPAP and non-CPAP groups after approximately 5 years (primary event rate ~20% in both arms), except when considering only those adherent to CPAP, where a benefit did emerge. Another trial, the Impact of Sleep Apnea syndrome in the evolution of Acute Coronary syndrome. Effect of intervention with CPAP (ISAACC 2020) trial [38] evaluated CPAP in patients with acute coronary syndrome who were found to have OSA; the results indicated no reduction in subsequent cardiovascular events with CPAP compared with usual care, although CPAP did improve daytime sleepiness and blood pressure.

Interventional studies on HF in patients with CSA have proven to be even more complex. The SERVE-HF trial [13,39] unexpectedly revealed an increase in cardiovascular mortality among patients with HFrEF treated with ASV for predominant CSA. The hazard ratio for cardiovascular death with ASV was 1.34 (95% CI 1.09–1.65), resulting in an absolute annual risk of 10% compared to 7.5% [39]. This finding was surprising, as smaller studies had suggested potential benefits of treating CSA, such as improving LV ejection fraction or quality of life. One hypothesis is that in some patients, eliminating protective ventilatory oscillations, which may enhance oxygenation but increase intrathoracic pressure and reduce preload, could worsen cardiac output. Alternatively, ASV devices may induce arrhythmias through inappropriate hyperventilation [40]. However, the exact underlying mechanism remains unclear. Subsequently, the ADVENT-HF trial [41] assessed ASV in a broader HFrEF with sleep apnea (either OSA or CSA) population and found no significant effect on either the composite outcome of death, life-saving cardiovascular intervention, or HF hospitalization, or on mortality alone, although ASV did effectively reduce the apnea-hypopnea index (AHI) and improve sleep parameters. Thus, currently, there is no evidence that treating CSA in HFrEF improves survival; in fact, it could be harmful in certain severe cases, leading current guidelines to advise against ASV in HFrEF with an LV ejection fraction of ≤45%. Conversely, CSA in HF is clearly a high-risk marker, as its presence often indicates advanced disease and portends a poor prognosis if the underlying HF is not aggressively managed.

These findings underscore that SDB is a significant comorbidity in CVD; however, simply addressing apnea with a “one-size-fits-all” approach has not proved effective in consistently preventing cardiovascular events. It is crucial to recognize that these neutral trials do not suggest that SDB is harmless to the heart. Rather, they highlight the need to refine the criteria for determining when and how we treat SDB in patients with cardiac disease as well as which patients should even be treated. Subgroup indications, such as the benefits of CPAP in adherent patients or those with very severe desaturation, imply that there may be a subset of patients with sleep apnea who do benefit from cardiovascular protection with treatment. Table 1 (Ref. [11,12,13,38,41,42]) provides an overview of the major clinical trials examining the impact of sleep apnea treatment on cardiovascular outcomes.

**Table 1.** **Key trials evaluating sleep apnea therapy and cardiovascular risk**.

Trial (Year)	Population	Intervention	Average usage	Primary outcome	Key findings
SAVE (2016) [12]	OSA (moderate-severe) + established CVD (MI, stroke, etc.), n = 2687, mostly asymptomatic (ESS score ~7)	CPAP + usual care vs. usual care alone	3.3 h/night	Composite CV events (CV death, MI, stroke, hospitalization)	No significant difference: 17.0% (CPAP) vs 15.4% (control) over ~3.7 years (HR 1.10, 95% CI 0.91–1.32, *p* = 0.34). CPAP improved sleepiness and quality of life but did not reduce CV events. Adherence was modest (3.3 h/night); post hoc analysis suggested benefits in those using CPAP >4 h/night.
RICCADSA (2016) [11]	Coronary artery disease + OSA (AHI ≥15 events/h) without daytime sleepiness (ESS score <10), n = 244	CPAP vs. no CPAP (open-label)	4.4 ± 2.3 (at 1 month) to 6.9 ± 1.2 (at 5 years) h/night*	Composite CV events (repeat revascularization, MI, stroke, CV death)	No overall benefit: Event rate ~18% (CPAP) vs 22% (control) in ~5 years, HR 0.80, *p* = 0.45. However, patients who were adherent (≥4 h/night) had a 71% relative risk reduction in events. This suggests that CPAP can be beneficial if used consistently, but routine prescription for non-sleepy OSA had no ITT benefit.
ISAACC (2020) (Spanish Multicenter) [38]	Acute coronary syndrome patients found to have OSA (moderate-severe) post-ACS, n = 1867	CPAP vs. usual care	2.78 h/night	Composite CV events (CV death, re-MI, stroke, hospitalization for HF)	No difference in cardiovascular events at ~3 years (16% vs 17%, *p* = 0.40). CPAP group patients had better daytime sleepiness and blood pressure control. However, adherence among patients with asymptomatic OSA was suboptimal.
SERVE-HF (2015) [13]	HFrEF (LVEF ≤45%) with predominant CSA (AHI ≥15 events/h, central ≥50%), n = 1325	Adaptive Servo-Ventilation (ASV) vs. control (no ASV)	3.7 h/night	All-cause death or life-saving CV intervention (primary); also CV death examined	Harm signal: ASV increased mortality. All-cause death: 34% vs 29% (HR 1.28, *p* = 0.01); CV death: 10% per year (ASV) vs 7.5% per year (control). Trial stopped early. No improvement in HF hospitalizations or symptoms with ASV. Result led to contraindication of ASV in HFrEF with LVEF ≤45% due to safety.
ADVENT-HF (2024) [41]	HFrEF with sleep apnea (OSA or CSA, AHI ≥15 events/h), n = 872 randomized	ASV vs. optimal medical therapy (control)	3.8 h/night	Composite of death from any cause, life-saving CV intervention (transplant/VAD), or unplanned hospitalization for HF	No significant benefit or harm: The primary composite outcome was similar between the ASV and control groups (event rate ~28% vs 32%, *p* = NS). All-cause and CV mortality were not different. ASV effectively reduced AHI and improved sleep metrics, but this did not translate into improved clinical outcomes (The trial excluded patients with very low EF (<25%) initially, and OSA patients with ESS score >10 were treated with CPAP, focusing analysis on CSA/low-risk OSA).
Others (e.g., CANPAP 2005, etc.) [42]	(For brevity, smaller trials and observational studies were omitted from this summary)	–	–	–	Earlier trials like CANPAP (CPAP in HF with CSA) hinted at improved EF but no survival benefit. Observational data suggest treating OSA may reduce atrial fibrillation recurrence and improve blood pressure, but randomized clinical trial evidence for hard outcomes is lacking outside those subgroups.

ESS, Epworth Sleepiness Scale; MI, myocardial infarction; CV, cardiovascular; HF, heart failure; EF, ejection fraction; HFrEF, heart failure with reduced ejection fraction; LVEF, left ventricular ejection fraction; OSA, obstructive sleep apnea; CSA, central sleep apnea; ASV, adaptive servo-ventilation; AHI, apnea-hypopnea index; ITT, intention-to-treat analysis; VAD, ventricular assist device; SAVE, Sleep Apnea Cardiovascular Endpoints; RICCADSA, Randomized Intervention with CPAP in Coronary Artery Disease and Obstructive Sleep Apnea; ISAACC, Impact of Sleep Apnea Syndrome in the evolution of Acute Coronary Syndrome; SERVE-HF, Treatment of Predominant Central Sleep Apnea by Adaptive Servo Ventilation in Patients with Heart Failure; ADVENT-HF, Adaptive servo-ventilation for sleep-disordered breathing in patients with heart failure with reduced ejection fraction; CANPAP, The Canadian Continuous Positive Airway Pressure for Patients with Central Sleep Apnea and Heart Failure. *In the RICCADSA trial, 40.2% (49/122) of patients in the CPAP group returned their devices within 2 years. Reported CPAP usage values represent cross-sectional means calculated at each follow-up time point.

Identifying these patients represents a key area for innovation, where ML can contribute by analyzing extensive patient datasets to uncover predictive patterns. In the following sections, we explore how AI has been utilized in this field and the potential benefits it may offer.

## 5. AI Applications for SDB and Cardiovascular Health

In recent years, AI and ML techniques have swiftly made their way into the realms of cardiology and sleep medicine, leveraging the abundance of available digital health data ranging from high-resolution physiological signals to imaging and electronic health records (EHRs). AI applications can be categorized into three main areas within the scope of sleep apnea and CVDs: (1) detection and diagnosis of SDB, (2) risk prediction and outcome prognostication for patients with SDB, and (3) therapeutic guidance and management.

### 5.1 AI for Detection and Diagnosis of Sleep Apnea

One of the most pressing needs is the development of improved screening and diagnostic tools for OSA, as the vast majority of cases remain undiagnosed [43]. The gold-standard diagnostic test, overnight polysomnography (PSG), is resource-intensive and requires specialized sleep laboratories, multiple sensors (electroencephalography [EEG], electrocardiography [ECG], oximetry, airflow, respiratory effort, etc.), and expert scoring. AI algorithms have been used to automate the analysis of standard sleep study data or to facilitate OSA detection using more easily obtainable signals or clinical data [44]*.*

#### 5.1.1 Clinical and Anthropometric Data

Even without specialized sensors, ML can leverage routine clinical variables—such as age, sex, body mass index (BMI), neck circumference, blood pressure, and questionnaire responses to predict the likelihood of OSA. While conventional risk scores such as the STOP-Bang questionnaire exist, AI could be developed to identify nonlinear patterns across numerous variables. For instance, logistic regression and decision-tree models that utilize demographic and anthropometric features have achieved an accuracy of approximately 85–95% in classifying patients with OSA [45,46]. One study highlighted a straightforward decision-tree model that, by using only age, sex, and systolic blood pressure, could identify OSA with approximately 97% accuracy in their cohort [46], demonstrating its potential when optimal cut-offs are derived from data. More commonly, models incorporating 10–20 features—including neck size, snoring history, and comorbidities—have achieved areas under the curve (AUCs) of around 0.80–0.90 for predicting moderate-to-severe OSA [47,48,49,50,51], surpassing standard tools in primary care. Such models can be integrated into EHR systems to identify high-risk patients for definitive testing.

#### 5.1.2 Wearable and Sensor Data

A promising area of AI in OSA diagnosis involves the analysis of physiological signals obtained from wearables or simpler devices. For example, ML-based analysis of nocturnal oximetry can reliably detect moderate to severe OSA by capturing cyclical desaturation and pulse-rate patterns. Some models have achieved sensitivity and specificity of >90% using oximetry alone [52,53]. In one study, a model employing a random forest on oximetry features reported an AUC of approximately 0.90 and an accuracy of approximately 90% [53], exceeding those of many multi-channel screening devices. Similarly, ECG or heart-rate variability signals contain OSA signatures, such as episodes of bradycardia during apnea followed by tachycardia upon arousal, known as cyclic variation in heart rate. Neural network models trained on single-lead ECG (e.g., from a night-long Holter test or even a smartwatch) have been reported to detect OSA with accuracies in the 80–90% range [54,55,56,57,58,59,60,61], and the FDA has even approved some algorithm-driven devices for detection. Additionally, snoring and breathing sounds recorded via smartphones or bed-mounted sensors can be analyzed using AI to screen for OSA with over 90% accuracy by identifying characteristic snore acoustics and breathing pauses [62]. These contactless approaches are especially promising for population screening.

#### 5.1.3 Imaging and Anatomical Analysis

Anatomical factors, such as retroglossal airway volume, tongue size, and craniofacial structure, contribute to OSA. AI has been used to analyze imaging data (cephalometric radiography, computed tomography [CT], and magnetic resonance imaging [MRI] of the upper airway) to predict the presence of OSA. A notable instance involved the use of thoracic CT scans, analyzed using a deep learning algorithm (Faster R-CNN), to identify differences in airway morphology in patients with HF and comorbid OSA [63]. The AI model accurately segmented the airway structures and detected significantly greater collapsibility in the retropalatal and retroglossal regions of patients [63], achieving approximately 96.6% accuracy in distinguishing OSA from non-OSA [63]. This indicates that routine imaging, such as a chest CT performed for another reason, could be repurposed by AI to flag potential sleep apnea. This is particularly relevant in cardiology, where many patients undergo CT scans for other purposes. Even facial photography with AI analysis has been explored, as certain craniofacial phenotypes, such as maxillary narrowing and jaw position, correlate with OSA, demonstrating moderate predictive value [64,65].

#### 5.1.4 Automatic PSG Analysis

AI is increasingly utilized in sleep laboratories to automate the scoring of polysomnograms and the detection of apnea/hypopnea events, arousals, and even sleep stages. Deep learning models can analyze raw multi-channel PSG data to identify apneas with a high level of agreement to human scoring, significantly reducing the labor involved [58,66,67,68,69,70,71,72]. While this application is generally within the field of sleep medicine, it intersects with cardiology when considering that automated analysis can help scale up diagnostics and potentially identify subtle respiratory events linked to arrhythmias or nocturnal ischemia that humans may overlook. Automated scoring tools have demonstrated apnea detection accuracies that often exceed 90%, and some have been FDA-cleared for clinical use [73,74]. These technologies are summarized in Table 2 (Ref. [75,76,77,78,79,80,81]).

**Table 2.** **Overview of AI-enabled technologies for sleep apnea**.

Product name	Company/Country	Year	Core AI mechanism	Application
EnsoSleep [75]	EnsoData, United States	2021	Deep learning (CNN + LSTM) algorithms trained on multi-channel PSG to automatically detect sleep stages, respiratory, arousal, and movement events.	AI-assisted scoring software for clinical polysomnography.
SleepCheckRx [76]	ResApp Health, Australia	2022	Locked ML analysis of breathing and snoring sounds recorded via smartphone microphone.	App-based home prescreening for OSA risk in adults.
Belun Sleep System [81]	Belun Technology, China	2023	AI software (“Belun Sleep AI”) analyzes PPG and accelerometer signals from a finger-worn ring to estimate apnea–hypopnea index (bAHI) and sleep stages.	Home screening for sleep apnea.
SOMNUM [78]	Honeynaps Co., Korea	2023	Deep learning–based automated algorithms trained on PSG data to detect sleep stages, arousal, respiratory, and limb movement events.	AI-assisted scoring software for clinical polysomnography.
SANSA [77]	Huxley Medical, United States	2024	Cloud-based algorithm combining signal processing and AI/ML components to analyze PPG, ECG, and accelerometer data from a chest-worn patch.	Home testing for sleep apnea.
AcuPebble Ox (200) [79]	Acurable Limited, United Kingdom	2025	AI analysis of respiratory and acoustic signals from a small neck-worn sensor and PPG sensor to extract OSA parameters.	Home screening for sleep apnea.
TipTraQ [80]	PranaQ Pte. Ltd., Singapore	2025	Cloud-based AI analysis of PPG and accelerometer signals from a finger-worn sensor to estimate AHI, ODI, TST, and REM sleep time.	Home testing for sleep apnea.

CNN, convolutional neural network; LSTM, long short-term memory; PPG, photoplethysmography; ODI, oxygen desaturation index; REM, rapid eye movement; TST, total sleep time; ECG, electrocardiogram.

Overall, the integration of AI for OSA diagnosis holds promise for improving early detection. By lowering the barrier to diagnosis, many more patients with significant OSA could be identified and referred for therapy. This is crucial because untreated OSA in a cardiac patient can exacerbate their disease. High-sensitivity screening models can ensure that fewer high-risk patients remain undiagnosed.

### 5.2 AI for Risk Prediction and Prognosis in Sleep Apnea Patients

Beyond merely identifying the incidence of sleep apnea, a crucial question—particularly for cardiologists—is determining which patients with sleep apnea are at the highest risk for cardiovascular complications. Not all individuals with OSA or CSA develop heart disease or experience cardiac events; some may follow a benign course, whereas others may be on a path toward stroke, arrhythmias, or exacerbation of HF. Traditional risk stratification metrics, such as the AHI, oxygen desaturation indices, and symptom burden, do not fully capture risk—as an example, patients with similar AHI values can exhibit significantly different hypoxic or hemodynamic stress profiles. AI models can incorporate multidimensional data to predict sleep apnea outcomes more accurately than any single metric. These models often integrate features from sleep studies with clinical data.

#### 5.2.1 Predicting Incident Cardiovascular Events

In a notable study by Silva et al. [82], researchers utilized an EHR-based dataset with data for over 10,000 individuals, both with and without OSA, AF, and CAD, to train ML models aimed at predicting new-onset AF, with a particular focus on the contribution of OSA. Clustering and k-nearest neighbors analysis revealed that OSA was a significant predictor, with affected individuals exhibiting approximately a 1.5-fold higher risk of AF (HR ~1.54, 95% CI 1.22–1.94) after adjustment [82]. The ML model identified OSA, hypertension, coronary disease, and chronic kidney disease as the key risk factors for AF. OSA also clustered with hypertensive and CAD phenotypes, highlighting its role in cardiovascular multimorbidity and the necessity for increased arrhythmia vigilance in these patients. Another example involves ML classifiers used to predict major adverse cardiac events (MACE) in patients with OSA based on their polysomnographic features and laboratory results [83]. Some early studies (e.g., using neural networks on PSG data) [84,85] demonstrated improved risk prediction compared to using AHI alone. More recently, advanced deep learning approaches that leverage raw PSG signals have further enhanced cardiovascular risk prediction. For example, He et al. [86] developed an interpretable framework that achieved strong predictive performance for cardiovascular outcomes, including AF (AUC 0.965), HF (0.861), and CVD mortality (0.854). Large-scale foundational models such as SleepFM have demonstrated the ability to predict a broad spectrum of diseases using a single night of sleep data [87]. These findings highlight the growing potential of PSG-based AI models to move beyond traditional indices and enable comprehensive cardiovascular risk profiling. Selected representative AI models applied to SDB and related cardiovascular outcomes are summarized in Table 3 (Ref. [46,52,56,63,82,86,87,88,89,90,91,92,93,94,95]).

**Table 3.** **Selected AI approaches for SDB and cardiovascular outcomes**.

Study (Year)	Application	Data inputs	AI approach	Key results
**Detection and diagnosis**				
Ting et al., 2014 (J Med Syst) [46]	Screening for moderate to severe OSA based on clinical variables	EHR records of 540 patients (61 in the non-OSA group, 85 with mild OSA, 131 with moderate OSA and 263 with severe OSA cases), using three non-invasive features (age, sex, and average SBP)	Integrate an expert-based feature extraction technique using decision tree algorithms	Decision tree model achieved 96.9% accuracy, 98.2% sensitivity, 93.2% specificity for detecting moderate/severe OSA, showing that clinical variables can be used effectively to screen for moderate/severe OSA.
Erdenebayar et al., 2019 (Comput Methods Programs Biomed) [56]	Automatic detection of sleep apnea events	ECG recordings segmented (10 s windows) from 86 patients	Deep learning model (CNN, RNN) for automated apnea/hypopnea detection using ECG data	The best model achieved 99.0% accuracy and 99.0% recall in distinguishing apnea and hypopnea events, demonstrating that deep learning can effectively extract temporal features from ECG for automated OSA event detection.
Tian et al., 2022 (Comput. Math Methods Med.) [63]	OSA detection via imaging in patients with HF	Thoracic CT scans of patients with HF (30 with OSA, 30 without OSA); airway morphology features	Deep learning (Faster R-CNN) for airway segmentation and classification	CT-based AI model detected OSA vs non-OSA with 96.6% accuracy. Found significantly greater airway collapse in retropalatal and retroglossal regions in OSA group (*p* < 0.05). Demonstrates feasibility of anatomical AI screening for OSA in cardiology patients.
Du et al., 2022 (Antioxidants) [91]	Identifying metabolic and inflammatory biomarkers in patients with hypertensive and OSA	Plasma metabolomics & lipidomics profiling of 559 patients. OSA diagnosed via PSG, and clinical indicators (BP, BMI, etc.)	ML models: LASSO, random forest, SVM for feature selection & classification	Identified distinct metabolic signatures in patients with hypertension and OSA. Elevated oxidative stress markers (e.g., uric acid, oxidized lipids). Elevated inflammatory mediators. The ML model classified OSA vs non-OSA with high accuracy (AUC >0.90). This suggested that metabolic fingerprinting may aid in non-invasive OSA detection.
Levy et al., 2023 (Nat Commun.) [52]	Automated diagnosis of OSA using oximetry data	Single-channel nocturnal oximetry data from 12,936 subjects across multiple cohorts	Deep learning model (OxiNet): hybrid CNN + CRNN architecture integrating temporal and clinical features	Achieved AUC = 0.96 for detecting moderate-to-severe OSA; validated across independent datasets and demographics, supports scalable, low-cost AI-based OSA screening.
Giorgi et al., 2025 (Systematic Review, Healthcare) [92]	AI for OSA screening – review of 65 studies	Various (anthropometric data, oximetry, ECG, audio, etc.) for 109,000 patients	Meta-analysis of ML classifiers (LR, SVM, NN, etc.)	AI models improved OSA detection with sensitivity/specificity often >90% vs standard tools. Example: an MLP classifier using neck circumference, BMI, etc. had 86% accuracy; an SVM using optimized features had 80% accuracy (vs 73% for logistic regression). Oximetry-based AI achieved AUCs of ~0.89 and 90% accuracy. Suggests that AI screening can substantially reduce missed OSA cases.
**Risk prediction and prognosis**				
Silva et al., 2022 (Front. Cardiovasc. Med) [82]	Predicting AF risk in OSA (and CAD) – epidemiologic ML study	EHR records of 22,302 patients (10k with AF/CAD/OSA); Features: diagnosis of OSA, CAD, HTN, CKD, age, sex over 9 years	K-means clustering; k-NN classifier; Cox survival ML model	Identified OSA as a significant predictor of new-onset AF. Patients with OSA had a 1.5× higher hazard of AF (HR ~1.54). An ML model combining OSA, CAD, HBP, and CKD achieved high accuracy (0.98) in classifying risk. Clustering showed OSA clusters with HTN and CAD in multimorbidity. Highlights the role of OSA in AF genesis.
Agrawal et al., 2023 (Ann Am Thorac Soc) [93]	Compare mortality risk in CSA vs OSA among U.S. veterans	Retrospective EHR data from 1,487,353 OSA and 2961 CSA patients in the U.S. Veterans Health Administration (2005–2018)	“Big data” Cox modeling; feature importance via ML	CSA was associated with higher mortality than OSA (adjusted HR 1.53). HF was the strongest predictor of early death in CSA (HR ~7.4 with HF vs without). ~20% of CSA patients died within 5 years of diagnosis. Highlights the need for aggressive management.
de Gonzalo-Calvo et al., 2023 (J. Transl. Med.) [89]	Phenotyping HF+CSA by microRNAs (risk stratification)	Plasma microRNA profiling of 587 patients with HF and CSA (from the SERVE-HF trial) as well as clinical data	Decision tree ML integrated with Cox regression	miR-133a-3p identified a low-risk subset: Patients with high miR-133a-3p had significantly lower event rates (death/HF hospitalization). The ML-derived decision tree showed that miR-133a-3p, combined with clinical factors, segregated a group with very low risk not captured based on NT-proBNP or clinical score. Suggests that biomarker-informed ML can refine risk stratification in CSA.
Kim et al., 2025 (J. Clin. Neurology) [88]	Mortality prediction in OSA patients (10- & 15-year)	Sleep study metrics (AHI, ODI, arousal index, etc.) + HRV during different sleep stages; OSA (n = 1790) vs. non-OSA groups (n = 1880)	Gradient boosting machine (LightGBM); plus survival analysis (Cox, KM curves)	ML model achieved AUC ≈ 0.806 for predicting 10–15 y all-cause mortality. Identified features: patients with higher vagal HRV during sleep had higher mortality (counterintuitively). The model stratified patients into high vs low risk groups with significantly different survival curves. Demonstrates that analyzing detailed sleep physiology via ML can yield prognostic insight beyond AHI.
He et al., 2026 (Sleep) [86]	Interpretable PSG-based framework for cardiovascular risk profiling	Raw sleep signals (EEG, ECG, and respiration)	Self-supervised multimodal deep learning + projection-score–based logistic regression	Improved prediction of cardiovascular outcomes beyond traditional risk factors (e.g., AF: 0.965, HF: 0.861, CVD mortality: 0.854), with strong potential for clinical integration.
Thapa et al., 2026 (Nat Med) [87]	Multi-modal PSG-based disease prediction	Multi-modal PSG signals (ECG, EEG, airflow, SpO_2_, etc.) from ~65,000 participants (>585,000 h across multiple cohorts)	Contrastive learning approach	Predicted >130 diseases from one-night sleep (C-index ≥0.75; all-cause mortality 0.84, MI 0.81, HF 0.80, AF 0.78); scalable, label-efficient analysis and disease prediction from sleep data.
**Therapeutic guidance and clinical management**				
Eguchi et al., 2022 (Sci Rep) [90]	Predicting poor CPAP adherence	CPAP usage logs + clinical features from 219 patients with OSA	Logistic regression and pairwise learn-to-rank (LTR) ML model for adherence prediction	Models achieved ~86.4% accuracy in identifying low-adherence users (<4 h/night); the key predictors were mask leak, pressure stability, and usage duration, highlighting the value of ML for improving CPAP adherence.
Mosteiro-Añón et al., 2025 (npj Prim Care Respir Med) [94]	AI-driven clinical decision support	Clinical and demographic data from 5385 patients with suspected sleep apnea	K-prototypes clustering + elbow method + silhouette analysis + random forest–based ICDSS	Identified 5 distinct clinical clusters with significant differences in AHI and CPAP indications; ICDSS achieved AUC 0.87–0.95 for assigning new patients to clusters, enabling early phenotyping and personalized diagnostic and treatment strategies.
Mount Sinai project (in development), press release 2024 [95]	Predicting CVD risk & treatment response in OSA	Planned use of multi-modal data: imaging, genomics, sleep data from a large OSA cohort	AI/ML (unspecified, likely deep learning + survival models)	(Expected outcomes) – aimed to create models that predict which patients with OSA will develop CVD and which ones will respond to therapy. Seeking to personalize OSA management in cardiology via AI (This entry illustrates an ongoing research direction; results pending).

RNN, recurrent neural network; LightGBM, light gradient boosting machine; LASSO, least absolute shrinkage and selection operator; LR, logistic regression; SVM, support vector machine; NN, neural network; MLP, multi-layer perceptron; k-NN, k-nearest neighbors; CT, computed tomography; BMI, body mass index; AF, atrial fibrillation; CAD, coronary artery disease; CSA, central sleep apnea; HF, heart failure; CKD, chronic kidney disease; HTN, hypertension; SBP, systolic blood pressure; HBP, high blood pressure; CPAP, continuous positive airway pressure; ECG, electrocardiogram; EEG, electroencephalography; EHR, electronic health record; AUC, area under the curve; ICDSS, intelligent clinical decision support system.

#### 5.2.2 Mortality Risk Stratification

A recent 2025 study by Kim et al*.* [88] used an innovative approach by training a model on detailed sleep analysis features to predict long-term mortality in patients with OSA. They examined data from a substantial OSA cohort, focusing on features such as sleep architecture, arousal counts, and heart rate variability (HRV) metrics across various sleep stages. Employing a Light Gradient Boosting Machine (LightGBM) algorithm, they created a risk stratification tool for predicting 10-year and 15-year all-cause mortality with moderate accuracy (AUC ≈ 0.806) [88], which is quite impressive for such extended predictions and effectively distinguished between high- and low-risk groups in Kaplan–Meier analysis [88]. Notably, newer PSG-based deep learning models have also demonstrated strong performance in predicting mortality risk, such as SleepFM, which achieved a C-index of 0.84 for all-cause mortality prediction from a single-night sleep recording [87]. Interestingly, increased parasympathetic activity during sleep (high HRV), which is usually seen as protective—was linked to poorer outcomes, possibly due to exaggerated vagal rebounds following apneas. This type of tool could be highly beneficial in clinical settings, steering us towards personalized prognostication rather than a one-size-fits-all treatment approach for OSA.

#### 5.2.3 Integrating Biomarkers and Genetics

Recent research is exploring the integration of AI with biomarkers or omics data to enhance risk stratification. In a 2023 study, de Gonzalo-Calvo et al. [89] employed a decision-tree ML method to merge microRNA profiles with clinical variables in patients with HF and CSA using data from the SERVE-HF trial. They found that elevated plasma levels of miR-133a-3p identified a subset of patients with HFrEF+CSA with a significantly reduced risk of adverse outcomes [89]. Essentially, the algorithm developed a risk stratification tree, where one branch—those with miR-133a-3p above a specific threshold, experienced notably fewer deaths or hospitalizations for HF than anticipated based on their clinical profile [89]. This “low-risk subphenotype” would not have been detected using clinical predictors or natriuretic peptide levels alone [89]. The integration of novel biomarkers with AI could further refine prognostic stratification in SDB, as indicated by miRNA-based models that identify distinct clinical phenotypes [89]. In the future, AI-driven risk scores may incorporate proteomic or genomic data along with polysomnographic features to assess an individual’s risk profile more accurately.

#### 5.2.4 Predicting Treatment Response

Another aspect of prognosis involves determining whether a patient will respond to a specific therapy. AI has the potential to predict which patients will benefit from CPAP in terms of reducing blood pressure or decreasing AF recurrence. Some pilot studies have attempted to predict CPAP adherence by applying ML to psychosocial and symptom data, which is crucial because adherence influences outcomes [90,96]. Other studies have examined OSA features, such as arousal threshold or loop gain (which can be estimated from PSG), to predict whether CPAP will lower a patient’s blood pressure [97]. Although these studies are in the early stages, theoretically, an algorithm could identify a patient’s OSA as hypertension-mediated (characterized by a high sympathetic tone), suggesting that treating OSA would likely improve their blood pressure. Conversely, another patient’s OSA may have been confined to REM sleep with minimal desaturation, indicating a limited cardiovascular impact and thus less urgency for aggressive cardiac treatment.

As illustrated by the examples in Table 3, it is evident that AI can identify patterns or combinations of risk factors that enhance our ability to predict significant outcomes beyond the capabilities of traditional analyses. It is important to note that many of these models are still in the research stage. The next critical step involves prospective validation and integration into care.

### 5.3 AI for Therapeutic Guidance and Clinical Management

The role of AI in directing therapy for sleep apnea and its cardiovascular consequences is still evolving, yet several promising pathways are attracting attention.

#### 5.3.1 Personalized Treatment Selection

As previously discussed, not all patients derive the same level of benefit from therapies like CPAP. Currently, clinical decisions often follow a one-size-fits-all approach or rely on trial and error [98]. However, AI has the potential to transform this by analyzing large datasets to determine which patient profiles respond best to specific interventions, thereby predicting the most suitable therapy for each individual. For example, an AI model could recommend treatments based on a patient’s phenotype. If craniofacial features are predominant, an oral appliance may be suggested; if the patient exhibits high loop gain (unstable chemoreflex), oxygen therapy or a carbonic anhydrase inhibitor could be considered. In cases of central apnea, the decision may involve optimizing HF therapy or adding oxygen therapy or using a phrenic nerve stimulation device (such as the remedē system) to address the apnea. For patients with comorbid insomnia and sleep apnea (COMISA), addressing insomnia should be prioritized to improve treatment adherence and outcomes [99]. In addition to treatment selection, AI could also predict individual responses to specific therapies. A conceptual example involves the use of neural network models on baseline clinical and PSG data to predict blood pressure response to CPAP. Preliminary studies indicate that certain features, such as baseline sympathetic activation and specific gene polymorphisms, may predict a more significant blood pressure drop with CPAP [100,101,102]. An ML model can integrate these factors to provide more accurate predictions than those based on a single factor alone.

#### 5.3.2 Optimizing Device Therapy and Adherence

Modern CPAP and ASV devices are considered “smart”, as many incorporate built-in algorithms—typically deterministic rather than AI-based—to adjust pressures in response to events [103]. Nonetheless, there is potential for more advanced adaptive algorithms, such as those using reinforcement learning, to dynamically optimize pressure settings or ventilation parameters for each patient. To enhance adherence, some companies are exploring the use of ML on early CPAP usage data and patient feedback to predict which patients are likely to abandon therapy and allow for proactive interventions, such as education, changing the masks, or adding humidification [90,104]. This approach is similar to the use of AI for predicting medication non-adherence in other chronic diseases. Furthermore, AI could assist in monitoring treatment-emergent issues, such as detecting emergent central apnea on CPAP and automatically adjusting settings or suggesting a switch to ASV if necessary [103,105].

#### 5.3.3 Cardiovascular Risk Mitigation

AI could also play a crucial role in managing the overall care of patients diagnosed with SDB and CVD. Imagine an algorithm that continuously monitors a patient’s data—such as CPAP machine readings, weight, blood pressure, and possibly data from a wearable ECG device, and predicts an impending HF decompensation or AF recurrence, enabling early intervention. Some implantable cardioverter-defibrillators (ICDs) and pacemakers already track indicators of sleep apnea, such as nightly heart rate variability and impedance changes that suggest periodic breathing [106,107,108,109]. AI could process these data streams to detect CSA/OSA episodes more accurately and alert clinicians. For instance, an ICD may record that a patient has developed frequent nighttime Cheyne-Stokes respiration using thoracic impedance sensors [110], and AI could then integrate this information with trends in daytime activity and fluid weight to predict the risk of HF hospitalization, prompting an intensification of diuretics or therapy before a crisis occurs [111,112].

#### 5.3.4 Clinical Decision Support

In both cardiology and sleep medicine, AI can function as a decision support system. Recent studies have demonstrated the feasibility of AI-driven clinical decision support systems in sleep medicine. For example, Mosteiro-Añón et al. [94] applied clustering techniques combined with a random forest–based intelligent clinical decision support system (ICDSS) to classify patients with suspected sleep apnea into clinically meaningful phenotypes using pre-test clinical data [93]. The model achieved high accuracy (AUC 0.87–0.95) in assigning new patients to clusters, supporting early phenotyping and more personalized diagnostic and treatment strategies [94]. Another notable development in this area is a collaboration announced in 2024 involving Mount Sinai, the American Heart Association, and others, aimed at creating AI-powered models to identify cardiovascular risk and treatment response in patients with OSA [95]. This project is expected to leverage extensive datasets of patients with OSA who have known outcomes and responses to CPAP to develop algorithms that can guide clinicians. For instance, if a patient is unlikely to benefit from CPAP in terms of reducing cardiovascular risk, they may be directed towards alternative treatments or clinical trials for new therapies. Although results are not yet available, this initiative exemplifies the forward-thinking approach of integrating AI into therapeutic decision-making [95].

It is important to note that currently, no AI algorithm is the standard-of-care for guiding therapy for sleep apnea, as most remain under investigation. However, there have been related successes in trials in cardiology, such as AI-guided titration of blood pressure medications and AI recommendations for HF management [113,114,115]. As the evidence base expands, it is reasonable to expect that similar tools to emerge at the intersection of sleep and cardiology.

## 6. Translational Implications: Bringing AI into Cardio-Sleep Clinical Practice

The intersection of AI, sleep apnea, and cardiovascular medicine offers significant translational potential. If fully realized, this could greatly enhance patient care across various levels, from early detection to personalized treatment and outcome monitoring. Here, we outline the key implications and challenges of integrating these advancements into clinical practice.

### 6.1 Improved Screening and Awareness

Given the high prevalence of occult sleep apnea in patients with cardiac conditions [6], AI-based screening tools can enable cardiologists and primary care physicians to detect SDB at an earlier stage. For example, an EHR-based integrated risk calculator utilizing an ML algorithm could operate in the background and notify the clinician: “This patient with refractory hypertension and obesity has an 85% predicted risk of OSA.” Such a prompt could lead to the clinician conducting a straightforward home sleep test followed by appropriate treatment. Early detection ensures that patients receive therapy before years of untreated apnea, which causes cardiovascular damage. It could also alleviate the burden on sleep laboratories by determining which patients require a full PSG and which ones can be managed with home tests or empiric therapy. However, successful implementation will necessitate integration with clinical workflows and educating clinicians on interpreting AI results, addressing concerns about false positives, and over-reliance on algorithms.

### 6.2 Risk Stratification and Focused Care

Conversely, AI could help prevent both over- and under-treatment by stratifying risk. In current practice, treatment decisions often prioritize very sleepy patients for therapy because of safety concerns such as driving risks. Cardiovascular prevention may justify treating even asymptomatic OSA if the risk is high; however, not all patients with OSA fall into the high-risk category. An AI-derived risk score could identify, for instance, the top 20% of patients with OSA who account for 80% of cardiovascular events [88]. These patients could then be directed towards more aggressive management, ensuring CPAP adherence with rigorous support, tight management of blood pressure and lipid levels, and possibly even the prescription of experimental therapies, such as anti-inflammatory medications targeting OSA-induced inflammation. Meanwhile, patients with lower-risk OSA could be managed conservatively or simply for symptom relief. This type of *risk-based resource allocation* [116] is analogous to the use of risk scores in CVD (e.g., FRAX tool for fractures [117], and ASCVD risk estimator for statin-related decisions [118], which are known to enhance healthcare efficiency and effectiveness).

### 6.3 Tailoring Therapy and Monitoring

As previously discussed, AI could assist in determining the appropriate treatment for patients, such as choosing between CPAP, oral appliances, surgery, or implants, based on predicted outcomes. This may also suggest when to escalate therapy [15]; for instance, if a patient using CPAP remains at high predicted risk, possibly due to residual events or other factors, the AI may recommend adding an adjunct therapy, such as an antihypertensive to counter sympathetic activation, or enrolling the patient in a trial for anti-inflammatory therapy in OSA. Another future application involves closed-loop systems: a CPAP machine that not only adjusts pressure but also potentially administers a stimulant early in the morning if it detects lingering apnea-related sleepiness or a pacemaker that increases the nocturnal pacing rate if it detects a prolonged pause due to CSA, thereby preventing blood pressure dips [103]. These interventions would depend on algorithms that monitor physiology and intervene within safe limits.

### 6.4 Cross-specialty Collaboration

AI can act as a bridge between cardiology and sleep medicine, as the overlap between these fields is increasingly acknowledged, as evidenced by the rise in joint clinics and combined conferences on sleep and heart health [119]. An AI tool integrated into a cardiology clinic can suggest a referral to a sleep specialist when necessary, supported by using data. Conversely, an AI in a sleep lab can identify patients with potentially uncontrolled AF or bradyarrhythmias requiring cardiology input [59,60,82,120]. This synergy would enhance comprehensive care by treating patients holistically rather than in isolated segments. Training clinicians in both fields to interpret AI outputs relevant to the other domain will be beneficial, such as a cardiologist understanding an “oxygen desaturation burden score” from an algorithm or a sleep physician comprehending a “predicted 5-year AF risk” in a patient with OSA.

### 6.5 Enhanced Patient Engagement

AI-driven tools, particularly those integrated into smartphones or other patient devices, have the potential to actively involve patients in their own care [121]. For instance, a mobile app utilizing AI to analyze snoring or sleep quality could send alerts to patients with messages such as “You showed signs of severe apnea last night; consider using your CPAP” or “Your risk score is high; it is crucial to maintain these lifestyle changes”. When patients receive concrete data and personalized feedback, they may be more inclined to adhere to therapy. Incorporating gamification elements, such as users earning points for nights with CPAP usage or good sleep, could further enhance adherence by building on AI analytics [122]. However, ensuring privacy and data security is essential because these tools manage sensitive health information.

### 6.6 Population Health and Remote Monitoring

With the advent of wearables, such as watches equipped with SpO_2_ sensors and rings that detect pulse and movement, we now have abundant data on sleep patterns from the general population [123]. AI has the potential to transform this vast dataset into actionable epidemiological insights. For example, it can identify clusters of individuals with undiagnosed moderate OSA and guide them toward appropriate care. On a public health scale, AI could help prioritize interventions, such as determining which communities may benefit from mobile sleep clinics or blood pressure screenings linked to sleep apnea campaigns. In the realm of remote patient monitoring, for patients known to have both HF and sleep apnea, AI can continuously analyze data to potentially predict decompensations or arrhythmias, thereby enabling preventive outpatient measures and reducing hospitalizations [124].

### 6.7 Reducing Bias and Improving Equity

Notably, AI has the potential to mitigate some biases in current practices. Research indicates that women with OSA are often underdiagnosed, partly due to differences in their symptom presentations [125,126]. If a well-trained AI model can accurately identify the correct signals, it could be gender-neutral, thereby enhancing diagnosis in women. Similarly, various ethnic groups exhibit distinct craniofacial risk factors and prevalence [127]; AI trained on diverse populations could improve detection in those that are frequently overlooked. However, caution is necessary, because AI can perpetuate bias if the training data are skewed. Ensuring diversity in training datasets is crucial, and models should be tested across different demographics [128]. If implemented correctly, AI could standardize care by focusing on objective data, thus reducing the disparities caused by inconsistent clinical suspicion.

### 6.8 Economic Considerations

From a cost perspective, implementing AI in this field involves certain initial expenses, such as those for software, integration, and personnel training, but it has the potential to generate substantial long-term savings. Enhanced screening can lead to the treatment of patients before they develop expensive conditions, such as strokes or HF exacerbations, while more precise therapy targeting can prevent overtreatment in those unlikely to benefit. If AI can prescreen effectively, the need for unnecessary sleep studies could decrease; however, caution is necessary to avoid misclassification and missed diagnoses [129]. Payers are increasingly interested in funding digital health solutions that demonstrably reduce costs.

### 6.9 Explainability and Trust

One of the main challenges in integrating AI into clinical practice is establishing trust, as both doctors and patients need to have confidence in AI recommendations. In a field where decisions carry significant consequences, black-box models can pose a substantial obstacle. Consequently, there has been an increasing focus on explainable AI. For instance, if an AI identifies a patient as high-risk, it should provide a rationale, such as “due to severe oxygen desaturations and a high burden of PVCs at night”, to assist clinicians in understanding and responding appropriately [130]. For clinicians, suggestions from AI should be regarded as probabilistic decision support rather than definite diagnostic conclusions. Clinicians should consider three key aspects: the nature of the output (e.g., risk prediction or classification), the factors driving the prediction (e.g., important features), and the applicability of the model to individual patients. Additionally, AI recommendations should be integrated into established clinical pathways and interpreted within the context of clinical judgment. Ultimately, AI should serve as an adjunct to support, rather than replace, physician decision-making.

### 6.10 Translational Gap

Another critical issue is translational gaps, which involve shifting AI findings from retrospective data to prospective testing. For instance, if an AI model predicts who will benefit from CPAP, the definitive test is to apply that model prospectively and treat only those predicted to benefit, and then compare the outcomes with those of treating everyone. However, such trials are yet to be conducted. There is a risk that AI models could become self-fulfilling; if we only treat those predicted to benefit, we may never discover whether others could have benefited as well [131]. These complexities suggest that while AI will inform decisions, clinicians will still need to use their judgment.

### 6.11 Regulation and Validation

For these AI tools to be implemented effectively, they must undergo thorough validation and secure regulatory approval, such as those from the FDA in the US, and manufacturers must demonstrate their safety and effectiveness [132]. Additionally, ongoing re-validation is crucial, as AI models can “drift” over time due to changes in population characteristics or when applied in different settings from the ones in which they were initially trained [133]. For instance, an AI designed to assess OSA risk may require periodic retraining or local recalibration when introduced into a new hospital with distinct patient demographics. Both clinicians and regulatory bodies should insist on transparency; if a model fails—whether by not predicting an outcome or by providing an incorrect prediction that causes harm—there must be accountability and a corrective mechanism in place [134,135].

In summary, although the integration of AI into the relationship between sleep apnea and CVD has transformative potential, it must be pursued with careful consideration. The ultimate aim is to establish a learning health system where data from each patient’s experience, including sleep study, CPAP device usage, and cardiovascular outcome data, continuously inform and enhance AI models, thereby improving care for future patients.

**Fig. 4.**
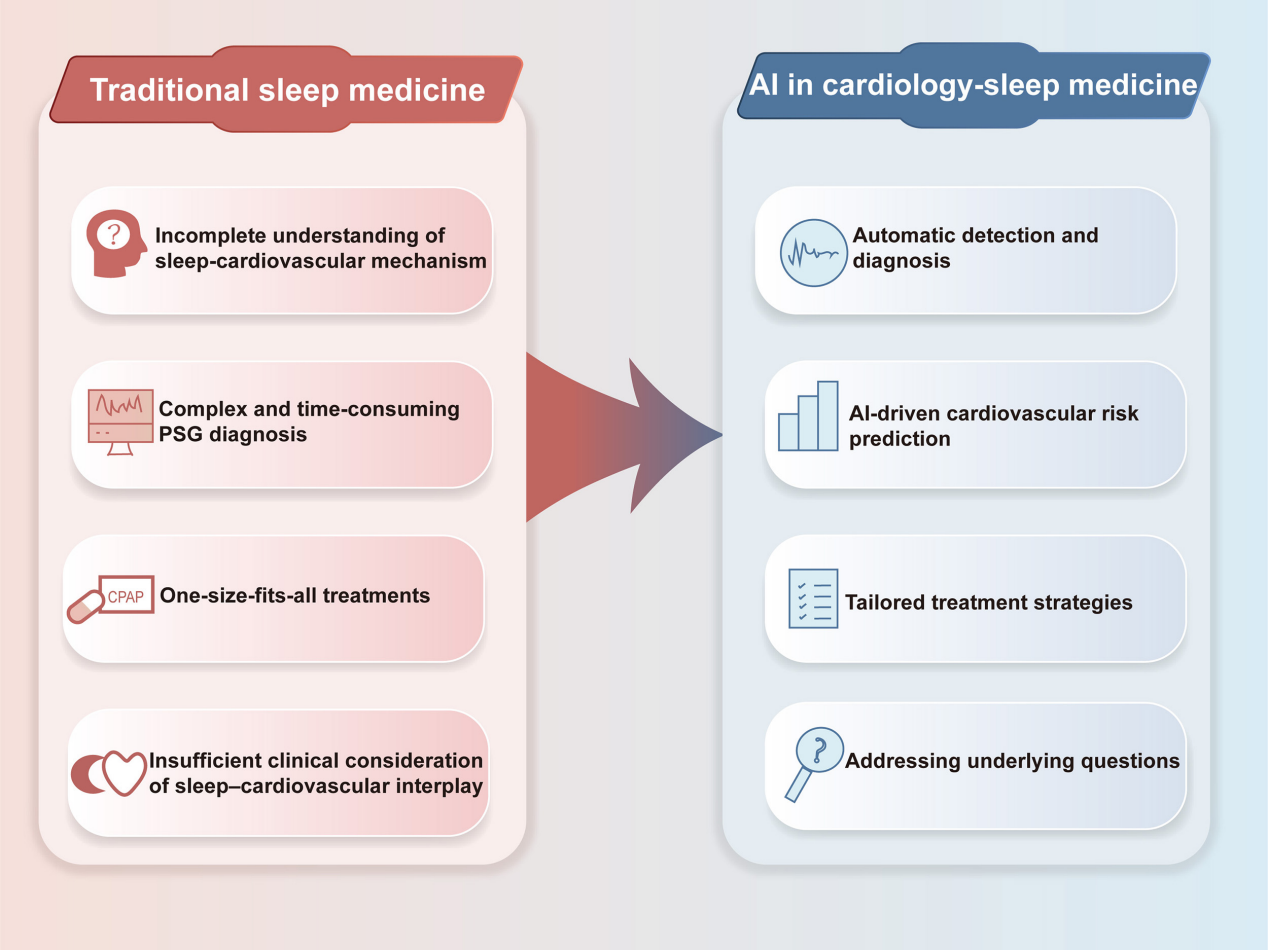
**The evolving landscape of cardiology–sleep medicine: from traditional approaches to AI-driven precision care**. AI could enable a shift from traditional, generalized sleep medicine to preventive and personalized cardiology–sleep care. Through automated detection, individualized risk stratification, and tailored therapy, AI can address key limitations such as mechanistic uncertainty and uniform treatment. The goal is timely diagnosis and risk-based management of SDB, supported—not replaced—by technology. Overcoming clinical, technical, and ethical hurdles will be essential, but AI holds promise for reducing CVDs by targeting modifiable contributors like sleep apnea.

These opportunities and challenges collectively reflect a broader shift toward a more integrated, preventive, and precision-oriented model of care. As illustrated in Fig. 4, the convergence of cardiology and sleep medicine through AI not only modernizes traditional pathways but also sets the foundation for a future in which individualized, risk-guided management of SDB becomes routine clinical practice.

## 7. Future Directions

In this section, we outline the key future directions, research needs, and challenges that must be addressed to fully leverage AI for enhancing cardiovascular outcomes in patients with sleep apnea.

### 7.1 Large-Scale Prospective Studies

Many AI models reviewed for OSA detection or risk prediction were developed using retrospective data, highlighting the need for prospective validation in real-world settings [133]. Screening tools should be tested in primary care centers to assess their effectiveness in increasing OSA detection and improving patient outcomes, and risk-stratification models need to be evaluated in trials comparing AI-guided care with standard care. Such interventional studies, potentially with cluster-randomized designs, are complex and demand multidisciplinary collaboration, but they will be crucial for establishing clinical utility before widespread adoption.

### 7.2 Refinement of Phenotypes and Endotypes

We anticipate a more in-depth exploration of OSA “endotypes”, which are subgroups defined by unique pathophysiological characteristics, such as upper airway anatomy dominance, loop gain dominance, or arousal threshold issues. AI, particularly when employing unsupervised learning techniques, such as clustering and latent class analysis, can assist in identifying these endotypes from large datasets and associating them with cardiovascular risk or treatment response. For instance, it has been proposed that a “hypoxemia-predominant OSA” endotype, characterized by prolonged oxygen dips and high oxidative stress, may present the highest cardiovascular risk and thus require more aggressive management [6,136]. Studies that integrate polysomnographic data with omics (genomics and proteomics) and detailed cardiovascular phenotyping (such as arterial plaque imaging) will be invaluable, essentially merging sleep medicine and cardiology data to uncover detrimental combinations [137,138,139,140]. One can envision future risk calculators that, by using not only AHI but also factors such as “hypoxic burden” (total area under the desaturation curve) and possibly a blood biomarker, provide a much more precise risk assessment for parameters like the 5-year risk of myocardial infarction [141,142]. Thus, AI models that incorporate such granular features are essential.

### 7.3 Technical Enhancements in AI Models

From a data science perspective, future AI models must address existing limitations. Many models have been developed in single-center settings; however, initiatives such as the National Sleep Research Resource are now aggregating thousands of sleep studies from diverse populations, and such data can be utilized to train more generalizable models [143]. Additionally, transfer learning may facilitate the adaptation of models across different cohorts, and it is crucial to maintain performance across various ethnic, age, and comorbidity groups to ensure equitable application [120,144]. Furthermore, AI models should incorporate time-series and longitudinal data more explicitly, as sleep apnea and cardiovascular parameters evolve over time. An ideal model would continuously learn from a patient’s trajectory using long-term data from wearable devices [124]. Advancing explainable AI approaches is also essential so that clinicians receive intuitive explanations (e.g., “patient’s risk is high due to X, Y, Z factors”) rather than a black-box risk score [135,145].

### 7.4 Integration of AI Into Devices

Future CPAP machines, ventilators, pacemakers, and wearables are likely to increasingly incorporate more AI technologies. For example, a CPAP device could detect a user’s breathing patterns and use a trained neural network to optimize pressure in a personalized manner, rather than relying on the current fixed algorithms. Similarly, a wearable ECG patch may continuously monitor both arrhythmias and OSA-related patterns, effectively allowing for a combined Holter and sleep test, with cloud-based AI algorithms interpreting the data. Regulatory approval for such smart devices will necessitate proven safety to ensure harm prevention if they fail (e.g., if an AI-driven CPAP device miscalculates the required pressure, there must be fallback limits). Nonetheless, the shift towards edge AI (real-time AI computation on the device itself) could improve responsiveness and patient autonomy [146]. The Internet of Things (IoT) in healthcare, where a patient’s various devices (CPAP, smartwatch, weighing scale, BP cuff) communicate with each other and are coordinated by AI, is a conceivable future paradigm that could help with comprehensive management of a patient’s condition on a daily basis [147].

### 7.5 Overcoming Clinical Adoption Barriers

Even the most advanced AI tool will prove to be ineffective if clinicians do not adopt it. Future research must focus on human factors: How can AI recommendations be presented in a user-friendly manner? What level of accuracy or certainty do clinicians need to trust AI for tasks such as deciding when to initiate therapy? Educating clinicians on the strengths and limitations of AI will be as crucial as the AI algorithms themselves [148,149]. In some instances, AI may operate in the background, assisting by, for example, automatically measuring sleep study indices and freeing up a clinician’s time. However, for critical decisions, building trust through explainability, validation, and even initially using AI only in an advisory capacity is prudent. There may also be medicolegal considerations—if AI suggests that a patient is at low risk and a negative outcome occurs, who is responsible: the provider or the tool? Therefore, clear guidelines and possibly regulatory frameworks must be developed [150].

### 7.6 Interdisciplinary Collaboration

The future of this field stands to gain significantly from enhanced collaboration among cardiologists, sleep specialists, data scientists, bioengineers, and industry professionals. Multi-center consortia that share data, while ensuring privacy safeguards, can facilitate the development of robust AI models. Competitions such as the PhysioNet challenge have already driven innovations in apnea detection using single-lead ECG; expanding these initiatives to include outcome prediction could further accelerate progress [151]. Additionally, involving patient advocacy groups, such as sleep apnea patient associations or HF support groups, in the process of designing AI tools will ensure that the patient perspectives, such as the usability of a home screening devices, are taken into account [152].

### 7.7 Addressing Underlying Questions

Notably, the application of AI could also help address fundamental scientific questions regarding sleep apnea and CVD [91,153]. For instance, by identifying features that most accurately predict outcomes, AI could suggest which mechanisms are more crucial. If an AI model determined that the duration of oxygen desaturation is a stronger predictor of stroke than the frequency of apneas, this would be pathophysiologically informative and suggest that sustained hypoxia may trigger coagulation more than brief dips. Similarly, if fluctuations in nighttime heart rate are strong predictors of arrhythmias, it highlights the significance of the autonomic pathway. Consequently, AI could help generate new hypotheses to be tested laboratory or clinical studies, fostering a beneficial cycle of discovery and clinical application [154].

## 8. Conclusion

In conclusion, the relationship between CVD and SDB involves a complex interplay of physiological factors, with AI poised to serve as a powerful orchestrator. Traditional methods have often struggled to translate mechanistic insights into improved outcomes, as evidenced by predominantly neutral results of previous clinical trials. These neutral results may be explained, at least in part, by suboptimal adherence, marked heterogeneity in SDB phenotypes and treatment response, overreliance on AHI-based classification, and delayed diagnosis after cardiovascular injury has become established. For example, COMISA represents a clinically important phenotype associated with higher cardiovascular risk, while insomnia symptoms may further compromise treatment adherence [99]. Most randomized clinical trials enrolled patients with a single phenotype (e.g., non-sleepy patients), who may be less likely to derive cardiovascular benefits from PAP therapy. The current classification system primarily relies on AHI, which does not adequately capture the underlying pathophysiological phenotypes, and phenotype-guided treatment strategies remain lacking. Evidence from murine models suggests that delayed intervention or sustained intermittent hypoxia may lead to persistent organ dysfunction that is not fully reversed by partial restoration of normoxia intended to simulate CPAP therapy [155].

However, we are now entering an era in which big data and AI could unravel such complexities by pinpointing which aspects of sleep apnea are most harmful to the heart, predicting which patients are at risk, and tailoring treatments accordingly. For example, recent AI models such as SleepFM have demonstrated the ability to predict a wide range of diseases—including HF and myocardial infarction, using multimodal PSG signals derived from a single sleep cycle [87]. Similarly, cluster-based ICDSS based on clinical clusters has been reported to enable early and accurate patient classification, supporting a precision medicine approach to sleep medicine [94].

Ultimately, the vision is one of preventive and personalized cardiology-sleep medicine, such that no patient with a dangerous sleep disorder goes undiagnosed, and those who are diagnosed receive management tailored to their unique risk profile, with AI aiding clinicians in making timely and informed decisions. Achieving this will require overcoming several technical, clinical, and ethical challenges, with AI positioned as an augmentative rather than a replacement tool. If realized, the convergence of AI with sleep and cardiovascular medicine could significantly enhance our ability to tackle two of the most pervasive health challenges of our time: heart disease and sleep disorders.
